# Intravenous Arginine Administration Promotes Proangiogenic Cells Mobilization and Attenuates Lung Injury in Mice with Polymicrobial Sepsis

**DOI:** 10.3390/nu9050507

**Published:** 2017-05-17

**Authors:** Chiu-Li Yeh, Man-Hui Pai, Yao-Ming Shih, Juey-Ming Shih, Sung-Ling Yeh

**Affiliations:** 1 Department of Nutrition and Health Sciences, Chinese Culture University, Taipei 11114, Taiwan; m8707008@hotmail.com; 2 School of Nutrition and Health Sciences, College of Nutrition, Taipei Medical University, Taipei 11031, Taiwan; 3 Department of Anatomy and Cell Biology, School of Medicine, College of Medicine, Taipei Medical University, Taipei 11031, Taiwan; pai0507@tmu.edu.tw; 4 Department of Surgery, Cathay General Hospital, Taipei 11073, Taiwan; yaomingshih2@gmail.com (Y.-M.S.); shihcvs@gmail.com (J.-M.S.); 5 Nutrition Research Center, Taipei Medical University Hospital, Taipei 11031, Taiwan

**Keywords:** arginine, sepsis, proangiogenic cells, Angpt/Tie-2, lung injury

## Abstract

This study investigated the influence of intravenous arginine (Arg) administration on alteration of circulating proangiogenic cells and remote lung injury in a model of polymicrobial sepsis. Mice were assigned to one normal control group (NC) and two sepsis groups that were induced by cecal ligation and puncture (CLP). One of the sepsis groups was injected with saline (SS), whereas the other (SA) was administered with a single bolus of 300 mg Arg/kg body weight via the tail vein 1 h after CLP. Septic mice were sacrificed at either 24 or 48 h after CLP, with their blood and lung tissues collected for analysis. Results showed that septic groups had higher proangiogenic cells releasing factors and proangiogenic cells percentage in blood. Also, concentration of inflammatory cytokines and expression of angiopoietin (Angpt)/Tie-2 genes in lung tissues were upregulated. Arg administration promoted mobilization of circulating proangiogenic cells while it downregulated the production of inflammatory cytokines and expression of Angpt/Tie-2 genes in the lung. The results of this investigation suggested that intravenous administration of Arg shortly after the onset of sepsis enhanced the mobilization of circulating proangiogenic cells, maintained the homeostasis of the Angpt/Tie-2 axis, and attenuated remote organ injury in polymicrobial sepsis.

## 1. Introduction

Sepsis is a systemic inflammation induced by severe infection that commonly occurs in critically ill patients. A recent finding suggested that during the clinical progression of a severe, uncontrolled infection, a continuum of severe sepsis to septic shock and ultimately to multiple organ failure (MOF) exists [1]. It has been speculated that the dysfunction of endothelial cells (ECs) may play a major role in the pathophysiology of sepsis-related MOF and that the degree of dysfunction has been suggested to be a predictor of mortality in sepsis [2]. Under physiological conditions, ECs provide an endothelial barrier to prevent vascular leakage and leukocyte migration into extravascular compartments. However, the excessive activation of the inflammatory responses during sepsis can alter the function of ECs, which consequently lead to an increase in vascular permeability, pro-coagulability, leukocyte adhesion and impairment of microcirculatory flow [3]. Thus, maintaining or restoring the normal function of ECs may be a novel approach for managing patients with severe infection and represents an attractive therapeutic target in cases of uncontrolled/refractory sepsis or septic shock [4,5]. 

Cumulative evidence has demonstrated that the repair of damaged vascular endothelium does not depend solely on the proliferation of local ECs. Bone marrow-derived endothelial progenitor cells (EPCs) may also play important roles in the process of angiogenesis and the re-endothelialization after vascular injury [6]. The percentage of circulating EPCs is low in normal physiological condition. However, EPCs increase significantly under conditions of metabolic stress, vascular injury, and inflammation [7,8,9]. A previous study revealed that an increased number of circulating EPCs was predictive of survival outcome in septic patients, which suggested the importance of EPCs in maintaining the integrity of vascular endothelium and restoring microcirculation during sepsis [10]. EPCs encompass two categories of cell types: the proangiogenic hematopoietic cells and the endothelial colony forming cells, both of which are involved in the process of angiogenesis [11]. Endothelial colony forming cells proliferate to form new blood vessels, whereas proangiogenic (hematopoietic) cells interact with local ECs to favor angiogenesis [11]. 

Although classified as a non-essential amino acid, l-Arginine (Arg) is an indispensable nitrogen carrier for the synthesis of urea, polyamines, proline, and other proteins in healthy adults [12]. In fact, it is considered semi-essential under stressed conditions such as trauma and sepsis [13,14]. Arg supplementation was originally discovered to have immune-regulatory properties [13] and was later demonstrated to be capable of attenuating inflammation [15]; thus, Arg is often used as a supplement for critical patients under high catabolic stress. Additionally, Arg is the substrate of nitric oxide (NO) synthase and the sole precursor of endogenous NO production [16]. A previous study found that Arg supplementation enhanced the expression of vascular endothelial growth factor (VEGF) and transforming growth factor (TGF)-β mRNAs in wound tissue of rats [17]. Finally, a study performed by Evrard et al. has shown that NO bioavailability and VEGF expression were critical for EPC mobilization and that TGF-β expression was found to increase the angiogenic property of EPCs [18]. The imbalance of angiopoietin (Angpt)/Tie-2 signaling is an accepted marker of vascular dyshomeostasis [19]. In this study, by identifying proangiogenic cells in mononuclear cells using specific markers, we hypothesized that Arg supplementation may promote proangiogenic cell mobilization and vascular endothelium maturation and thus alleviate remote organ damage. Since remote lung injury is the most frequently encountered complication in sepsis-associated MOF, the effects of Arg on systemic inflammatory response, Angpt/Tie-2 balance, and the histopathology of lungs were evaluated in a mouse model of polymicrobial sepsis. 

## 2. Materials and Methods

### 2.1. Animals

Eight-week-old male C57BL/6J mice weighing 20–22 g were purchased from National Laboratory Animal Center, Taipei, Taiwan at the beginning of the experiment. Mice were maintained on a 12-h day/night cycle in a temperature- and humidity-controlled room. Animal care and all experiments protocols were approved by the Institutional Animal Care and Use Committee at Taipei Medical University (LAC-2015-0018).

### 2.2. Experimental Procedures

Mice were randomly assigned to a normal group (NC, *n* = 8), a septic saline group (SS, *n* = 20), and a septic Arg group (SA, *n* = 20). There were no differences in the initial body weight (BW) among the three groups (data not shown). Sepsis was induced by cecal ligation and puncture (CLP) as described previously [19]. Briefly, mice were anesthetized with intraperitoneal (IP) injection of Zoletil (25 mg/kg BW) and Rumpon (10 mg/kg BW). A 1-cm midline abdominal incision was made with subsequent opening of the underlying peritoneum. The cecum was fully extracted from the peritoneal cavity and then ligated with 3-0 silk (Ethicon, Somerville, NJ, USA) at a level approximately 50% below the ileocecal valve. The distal cecum was punctured in a “through and through” manner using a 23-gauge needle. A small amount of fecal content was squeezed out and smeared onto the serosa of cecum. The punctured, fecal-coated cecum was then placed back into the peritoneal cavity and the laparotomy wound was closed in layers using 3.0 silk. Immediately after surgery, each mouse was resuscitated with sterile saline (40 mL/kg of BW) subcutaneously. One hour after CLP procedure, the SS group was injected with saline, while the SA group was treated with a single bolus of 300 mg Arg/kg BW given intravenously via tail vein. Mice were given buprenorphine (0.05 mg/kg BW) subcutaneously every 12 h for pain control and were euthanatized at either 24 or 48 h after CLP by cardiac puncture under anesthesia. Blood sample from each mouse was collected in heparinized tubes. Part of the whole blood collected was used for analyzing percentage of EPCs. The rest was centrifuged at 3000× *g* at 4 °C for 10 min to obtain the plasma, which was stored at −80 °C for further analysis. Lung tissues were removed and frozen at −80 °C for gene expression assays, but the right middle lobe of the lung from each animal was used specifically for histological analysis. 

### 2.3. Flow Cytometric Analysis Of Proangiogenic Cells in Blood

One hundred microliters of fresh blood were incubated with fluorescein isothiocyanate (FITC)-conjugated anti-mouse CD34 (RAM34, eBioscience, San Diego, CA, USA), allophycocyanin (APC)-conjugated anti-mouse CD309 (Avas12a1, eBioscience, San Diego, CA, USA), and phycoerythrin (PE)-conjugated anti-mouse CD133 (13A4, eBioscience, San Diego, CA, USA). After thirty minutes, lysing buffer (PharmLyse; BD Pharmingen, San Diego, CA, USA) was added to lyse the red blood cells (RBCs). Then the isolated proangiogenic cells were fixed using 2% paraformaldehyde before cytometric analysis. Mononuclear cells were first identified and CD34^+^/CD133^+^/CD309^+^-cells were gated. Flow cytometric analysis was carried out in accordance to standard settings on a multicolor BD FACS CantoII flow cytometer (BD Biosciences, San Diego, CA, USA), and data were analyzed with BD FACSDiva™ v6.1.3 software (BD Biosciences, San Diego, CA, USA) as described in the previous report [20]. We presented the value of proangiogenic cells in percentage instead of the absolute number among mononuclear cells because the number of proangiogenic cells in plasma are relatively low. In addition, cell loss may occur during the standard staining process. Therefore, in order to minimize the discrepancies between samples due to possible cell loss during the staining process, percentage of proangiogenic cells was calculated based on the number of mononuclear cells obtained from the same sample.

### 2.4. Measurements of Proangiogenic Cell-Mobilizing Factors in Plasma

The concentrations of C-X-C motif chemokine (CXCL) 12, matrix metallopeptidase (MMP)-9, VEGF, and tumor necrosis factor (TNF)-α were measured by Enzyme-Linked ImmunoSorbent Assay (ELISA) kits (eBioscience, San Diego, CA, USA). Known to be unstable in solution, NO was converted to stable nitrite and nitrate ions in aqueous solution. Using nitrate reductase, nitrate in the solution mixture was converted to nitrite of which the concentrations were measured with the Griess reagent. Plasma nitrite/nitrate concentrations were determined with a commercial kit (R&D Systems, Minneapolis, MN, USA) according to the manufacturers’ instructions.

### 2.5. Measurement of Cytokines in Lung Tissue

For each animal, a 20% lung homogenate was prepared by grinding 20 milligrams of lung tissue together with 100 microliters of ice-cold phosphate-buffered saline (PBS) using a homogenizer. The homogenate was centrifuged at 15,000 rpm for 20 min, and the supernatant was used for the analysis of cytokines. Concentrations of interleukin (IL)-1β, IL-6, TNF-α, IL-10, and TGF-β1 in lung tissue extracted supernatant were measured by ELISA kits (eBioscience, San Diego, CA, USA) according to the manufacturer’s instructions.

### 2.6. Angpt1, Angpt2, and Tie-2 Messenger (m)RNA Extraction and Analysis by Quantitative Real-Time Reverse-Transcription (RT) Polymerase Chain Reaction (PCR)

Total RNA was extracted from homogenized lung tissues using the Trizol reagent (Invitrogen, Carlsbad, CA, USA). The RNA pellet obtained was dissolved in RNase-free water and stored at −80 °C for further analysis. The concentration of RNA was quantified by a spectrophotometer with absorbance wavelengths set at 260 and 280 nm. Complementary (c)DNA was synthesized from total RNA using a RevertAid™ first-strand cDNA synthesis kit (Fermentas, Vilnius, Lithuania) according to standard protocols. Reverse transcription was performed by sequential incubation of the RNA and cDNA strand for 5 min at 65 °C, 60 min at 42 °C, and 5 min at 70 °C. Specific genes were amplified by a real-time RT-PCR using the 7300 Real-Time PCR System (Applied Biosystems, Foster City, CA, USA) with SYBR Green as the detection format. Primers that were used included the following: β-actin forward, ACC CAC ACT GTG CCC ATC TAC, and reverse, TCG GTG AGG ATC TTC ATG AGG TA; Angpt1 forward, GGG CAC ACT CAT GCA TTC CT, and reverse, GCG TCA GCT GCG AGT ACA TA; Angpt2 forward, CCA ACT CCA AGA GCT CGG TT, and reverse, CGG TGT TGG ATG ACT GTC CA; and Tie-2 forward, AGG CTA GTT CCA GGC TTG CTA A, and reverse, TGG AGA ACT TGG CAC AGG AAG A. All primers were purchased from Mission Biotech (Taipei, Taiwan) based on deposited cDNA sequences (GenBank database, NCBI). A total volume of 25 µL containing 1× Power SYBR Green PCR Master Mix (Applied Biosystems, Foster City, CA, USA), 400 nM of each primer, and 100 ng of cDNA were used in the amplification procedure. The proceeding reaction included: one cycle of 2 min at 50 °C and 10 min at 95 °C, followed by forty cycles of 15 s at 95 °C and 1 min at 60 °C, with a final dissociation curve (DC) analysis. Expression levels were quantified in duplicate by means of a real-time RT-PCR. Cycle threshold (CT) values for genes were normalized to β-actin and were used to calculate the relative quantity of mRNA expression.

### 2.7. Histopathology of Lung Tissue

From each animal, lung tissue of the right middle lobe was fixed with a 4% phosphate-buffered paraformaldehyde solution, dehydrated with a graded ethanol series, and embedded in paraffin. In order to determine the morphology of the tissue, series of 5-µm-thick sections were stained with hematoxylin and eosin (H&E). Digital images at 100× magnification were captured for each section and five fields per section were analyzed for morphological lesions. Images were assessed using an Image-Pro Plus software (Media Cybernetics, Silver Springs, MD, USA). A scoring system was used to grade the severity of lung injury [21] with some modifications. The score calculated for each animal was based on the following histological features: (1) atelectasis and interstitial edema; (2) alveolar edema; (3) perivascular hemorrhage and overdistension. Since specific immunostaining was not used to stain the lung tissues, the simplified histological parameters used for identifying the extent of lung injury among groups was less ambiguous and more objective when calculating the injury scores. Each feature was graded (0 = normal; 1 = mild; 2 = moderate; 3 = severe), and a total summed score can range from 0 to 9. 

### 2.8. Statistical Analysis

Data are expressed as the mean ± standard deviation (SD). All analyses were conducted using GraphPad Prism 5 (GraphPad Software, La Jolla, CA, USA). Differences among groups were analyzed by one-way analysis of variance (ANOVA) followed by Newman–Keuls multiple-comparison test. Differences within the same group at 24 and 48 h were analyzed by Student’s *t*-test. A *p* value of <0.05 was considered statistically significant. 

## 3. Results

There were no differences in the survival rate between the two septic groups at 24 h and 48 h after CLP (survival rate of 77% in both the SS and SA groups). 

### 3.1. Differences in Proangiogenic Cell Population in Blood after CLP

**Figure 1:**
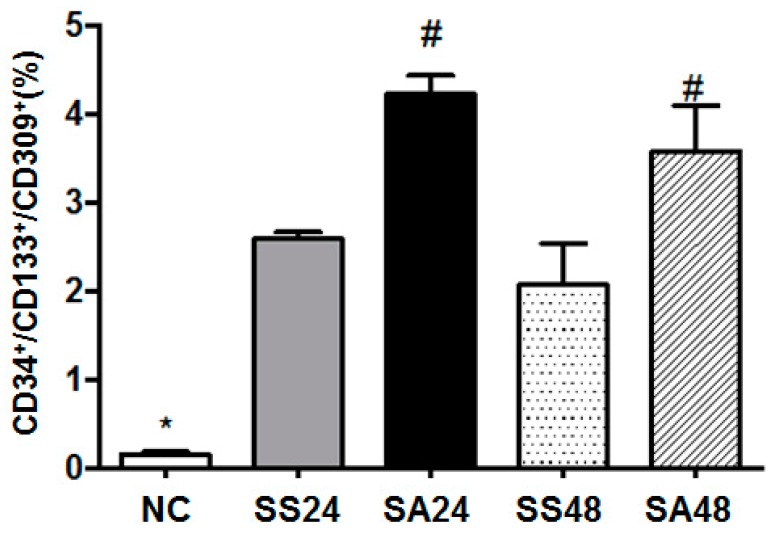
Distribution of circulating proangiogenic cells (CD34+/CD103+/CD309+) among the normal control (NC), sepsis with saline 24 h post-cecal ligation and puncture (CLP) (SS24), sepsis with arginine 24 h post-CLP (SA24), sepsis with saline 48 h post-CLP (SS48), and sepsis with arginine 48 h post-CLP (SA48) groups. All data are representative of duplicate measurements (*n* = 8). Data are presented as the mean ± SD. * Significantly differs from the sepsis group. # Significantly differs from the SS group at the same time point (*p* < 0.05).

The proportion of circulating proangiogenic cells increased significantly in all the sepsis groups. Moreover, the Arg-treated groups had even higher percentages of proangiogenic cells at both 24 and 48 h post-CLP when compared to that of the saline-treated groups at corresponding time points (Figure 1). 

### 3.2. Expression of Proangiogenic Cell-Related Proteins in Plasma

Compared to the NC group, protein concentrations of CXCL-12, MMP-9, VEGF, TNF-α, and NO were significantly higher in the sepsis groups. Both SA groups (SA24 and SA48) had higher levels of VEGF and NO whereas significant CXCL-12 elevation was observed only in the SA48 group. By contrast, levels of MMP-9 and TNF-α were significantly lowered when compared to those in the SS group at corresponding time points after CLP (Table 1).

### 3.3. Concentration of Cytokines in Lung Homogenates

The concentrations of IL-1β, IL-6, and TNF-α were significantly higher in the sepsis groups than those in the NC group. These pro-inflammatory cytokines were lower in the SA groups when compared to that of the SS groups. In contrast, the levels of anti-inflammatory cytokines, including IL-10 and TGF-β1, were significantly higher in the SA group than in the SS group, which was significant only at 24 h post-CLP (Table 2).

### 3.4. Expression of Angpt-1, Angpt-2 and Tie-2 mRNAs in Lung Tissues

**Figure 2:**
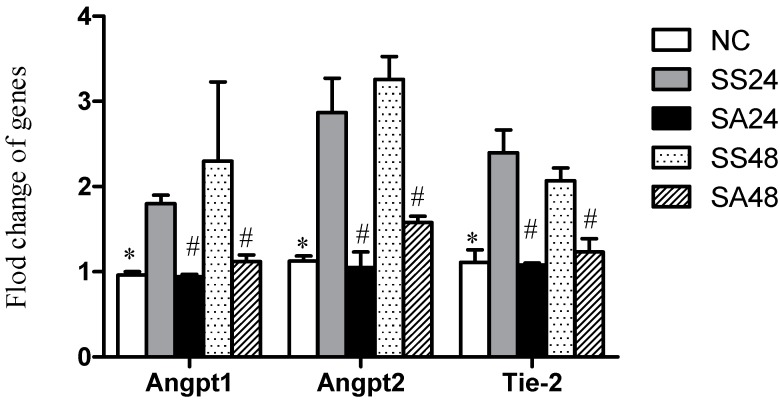
Expression of angiopoietin1 (Angpt1), Angpt2, and Tie-2 mRNAs in lung tissues. NC, normal control group; SS24, sepsis at 24 h with saline; SA24, sepsis at 24 h with arginine; SS48, sepsis at 48 h with saline; SA48, sepsis at 48 h with arginine. Data are presented as the mean ± SD. * Significantly differs from the sepsis group. ^#^ Significantly differs from the SS group at the same time point (*p* < 0.05).

The SS groups had higher expression of Angpt-1, Angpt-2, and Tie-2 mRNAs after CLP than those in the NC group. In general, the SA groups had significantly lower expression of the Angpt/Tie-2 genes than that of the SS groups at either time points. Furthermore, the degree of reduced expression of all three genes were comparable to that of the NC group (Figure 2).

### 3.5. Lung Histology

**Figure 3:**
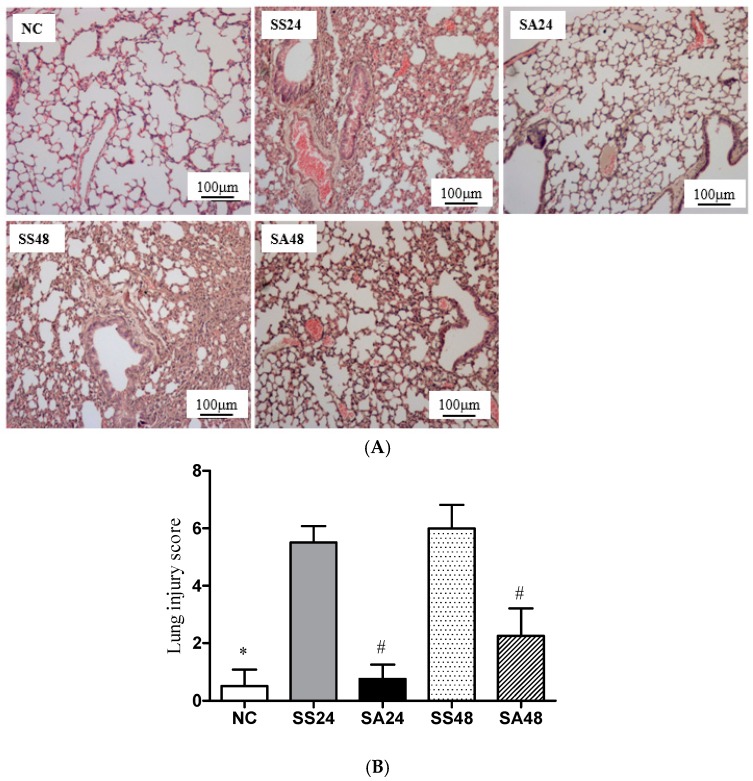
(**A**) Histopathology of the lung tissues; (**B**) Quantification of histological lung injury scores. All data are representative of duplicate measurements (*n* = 6). Data are presented as the mean ± SD. * Significantly differs from the sepsis group. ^#^ Significantly differs from the SS group at the same time point (*p* < 0.05).

The histopathological findings showed that the NC group had normal pulmonary architecture, whereas the groups that underwent CLP-induced sepsis resulted in thickening of the septal space. In addition, destruction of alveolar structures, neutrophil infiltration, edema, hyperemia, congestion, intra-alveolar hemorrhage, and scattered parenchymal debris were also observed. However, compared to the SS groups, the extent of inflammatory lesions within the lung alveoli was less severe in the SA groups at both time points (Figure 3A, SA24 and SA48). Semi-quantitative scores calculated according to the histological changes as described in “Methods” showed that the SA groups had significantly lower lung injury scores than that of the SS groups at either time point after CLP (Figure 3B).

## 4. Discussion

The use of arginine in critically ill patients has always been controversial, especially for septic patients. However, a study by Luiking et al. found that Arg infusion in sepsis stimulated NO production, reduced whole body protein breakdown, and was absent of any negative alterations in hemodynamic parameters [22]. A recent report concluded that Arg is safe and supplemental Arg infusion may be beneficial to septic patients [23]. In this study, we administered intravenously 300 mg Arg/kg BW shortly after onset of CLP-induced peritonitis. A previous study showed that intraperitoneal Arg pretreatment at a dose of 200 mg/kg BW attenuated liver damage in an ischemia/reperfusion rat model [24]. In general, mice have a higher metabolic rate than that of rats. Since we found that a single dose of 300 mg Arg/kg BW was able to reduce the level of inflammatory cytokines in plasma induced by ischemia-reperfusion injury from our preliminary study (unpublished data), this dosage was chosen for the experiment. In this study, the normal control (NC) group was served as the basal reference for comparison to the mice that underwent CLP-induced sepsis. We did not include a normal control group treated with Arg because the metabolism of Arg in sepsis had been previously reported. In a murine model of lipopolysaccharide (LPS) induced sepsis, plasma Arg and citrulline levels dropped significantly at 24 h after LPS induction [25]. In another rat model investigating the effect of LPS-induced sepsis on Arg, citrulline, and ornithine metabolism, arterial levels of Arg were reduced as early as 90 min after the onset of LPS sepsis [26]. Hence, in our study, Arg was administered immediately after the induction of CLP to account for the expected decrease in plasma Arg. In this study, we focused on whether Arg administration promoted EPCs under septic condition. Findings from the current study showed that Arg administration resulted in higher plasma NO levels, increased the percentage of blood proangiogenic cells, maintained the homeostasis of the Angpt/Tie-2 axis, and attenuated remote organ injury in condition of gut-derived polymicrobial sepsis. 

Sepsis is characterized by a systemic inflammatory response, which often leads to prominent disruption of microvascular endothelial structure and function during excessive and prolonged inflammation [27]. EPCs are particularly important in mediating the reparative process of damaged vascular endothelium and in maintaining endothelium homeostasis under catabolic conditions [28]. In this study, we found that sepsis results in a higher percentage of circulating proangiogenic cells. Also, the expression of proangiogenic cells mobilizing-related factors including CXCL-12, MMP-9, VEGF, TNF-α, and NO were all upregulated. CXCL-12, also known as stromal cell-derived factor (SDF)-1, promotes the mobilization and recruitment of EPCs [29]. MMP-9 has been found to induce the proliferation and mobilization of EPCs [30]. VEGF, an angiogenic factor, is responsible for the homing of EPCs [31]. It has been shown that the activation of MMP-9 in vascular endothelial cells involves the stimulation of VEGF receptors expressed on ECs by circulating VEGF in blood [30]. TNF-α is a commonly identified pro-inflammatory cytokine. In an ex vivo study, TNF-α was found to reduce the proliferation and migration of EPCs by decreasing the expression of SDF-1 mRNA and reducing the number of VEGF receptors, respectively [32]. NO has many physiological functions, one of which is involved in the mobilization of EPCs during the process of neovasculogenesis [33]. A study performed by Thum et al. showed that mobilization of EPCs via VEGF stimulation was reduced in an endothelial NO synthase-knockout mice model as compared to the wild type [34]. In this study, the higher proangiogenic cell percentage along with increased levels of NO, CXCL-12, and VEGF and decreased levels of TNF-α in blood after Arg administration suggested that Arg could enhance the release of EPC-mobilizing proteins, thus promoting the mobilization of proangiogenic cells. Arg is the substrate for both arginase and NO synthase (NOS). A pilot study by Tadie et al. [35] evaluated the effect of Arg supplementation on metabolic pathway in medical ICU patients. Their results showed that early enteral Arg administration increased ornithine synthesis, suggesting preferential use of the arginase pathway in these patients. In contrast, in a previous study investigating the differential expression of arginase and inducible NOS (iNOS) in the lungs in a CLP-sepsis rodent model, the authors demonstrated that strong expression of iNOS was found in alveolar, bronchial epithelial cell, endothelial cells, and alveolar macrophages, whereas arginase was almost undetectable after CLP [36]. Since patients in medical ICU are heterogeneous with arrays of underlying co-morbidities, the metabolic changes of these patients may be far more complex than the controlled CLP-induced peritonitis as represented in our model. We speculated that the single bolus administration of Arg may contribute to the observed increase in NO production possibly deriving from the citrulline/NO pathway.

Dysregulation of Angpt/Tie-2 signaling is a phenomenon observed in septic condition. Tie-2 is a transmembrane tyrosine kinase-type receptor specifically expressed on ECs and Angpts are ligands for Tie-2. It has been demonstrated that the activation of Tie-2 signaling in ECs favors blood vessel maturation and sustains the homeostasis of endothelial barrier function [19,37]. There are two types of Angpts: Angpt-1 is largely secreted by peri-endothelial cells, whereas Angpt-2 is mostly synthesized by the ECs [38]. Function of Angpt-2 was determined to act as a competitive antagonist of Angpt-1. A study demonstrated that the expression of Angpt-2 gene was upregulated but Angpt-1 gene was suppressed during sepsis, which correlated to the endothelial barrier dysfunction and consequent vascular leakages and vasculitis [37]. In this study, we found that the expression of Angpt 1 and 2/Tie-2 genes were upregulated in the saline groups, whereas this phenomenon was not observed in the Arg treated groups. Since Tie-2 signaling is known to stabilize endothelium barrier function [19], the elevated expression of Tie-2 gene observed with the saline groups may indicate that a more severe degree of vascular inflammation had occurred; the increased activation of Tie-2 signaling may reflect the severity of the damaged vascular barrier to undergo certain aspects of the reparative process. As expected in the lung tissues, we did find reduced concentrations of pro-inflammatory cytokines and increased concentrations of anti-inflammatory cytokines when Arg was administered. The histopathological findings were also consistent with the inflammatory status, implying that the remote injury to the lungs was less severe in the Arg treated groups. In this study, Angpt1, Angpt2, and Tie-2 were measured by real-time PCR to detect the expression of mRNA. Future studies are required to measure the actual protein concentrations of Angpt1 and Angpt2 ligands or Tie-2 using western blotting analysis.

Possible mechanisms responsible for the favorable effects of Arg on promoting proangiogenic cells mobilization and reserving Angpt-Tie2 homeostatic signaling axis may involve the participation of several complex regulations. First, a previous study concluded that the favorable effect of Arg may be a result of increased NO bioavailability [39]. An in vitro study by Lu et al. showed that the increased activity of eNOS and the amount of NO production had direct effects on the migration and proliferation of EPCs and their subsequent tube formation in the process of neoangiogenesis [40]. Second, an association between inflammatory status and EPC mobilization has been demonstrated. A previous study revealed that the presence of inflammatory cytokines promoted the apoptosis of EPCs; therefore, excessive pro-inflammatory response may be another crucial factor in reducing the number of circulating EPCs [32]. Arg was known to have an alleviating effect on inflammation; thus, its direct effect on reducing the production of pro-inflammatory cytokines may indirectly prevent apoptosis and impaired mobilization from occurring in the EPCs. The elevated NO production and reduced inflammatory response as observed in the Arg groups may be partly responsible for the enhanced mobilization of proangiogenic cells. We speculated that the attenuated inflammation might also provide a beneficial effect on maintaining more balanced Angpt-Tie2 signaling in remotely injured lung tissues when administered with Arg. However, further investigation is required to clarify whether NO plays a role and what mechanisms are involved in modulating the Angpt-Tie2 signaling axis in sepsis.

In this study, we did not observe a difference in mortality among groups. There are different factors that determine the mortality of CLP: the species of the animal, resuscitation, length of the cecal ligation, and the needle size used. In a previous study with 50% cecum ligated below the ileocecal valve, punctured with 23-gauge needle, and deprived of antibiotics treatment, the survival rate was estimated 77% at 24 h [41], which is identical to this study. According to the classification of sepsis, 50% cecal ligation and the use of a 22-gauge needle puncture was classified as “mid-grade” [42]. With this grade of sepsis, comparing the mortality between groups may not be easy. There are limitations in this study. Since an Arg control group was not included, the influence of Arg on proangiogenic cell alteration at baseline versus in sepsis model cannot be found. Additionally, the possible impact of IV Arg used prophylactically and different Arg dosages on sepsis may also be worthy of further exploration. 

## 5. Conclusions

To our knowledge, this is the first study to investigate the effect of Arg administration on the mobilization of proangiogenic cells and the alteration of Angpt/Tie2 homeostasis in sepsis. The findings of this study revealed that a single intravenous injection of Arg after CLP enhanced the release of CXCL-12, VEGF, and NO, which consequently resulted in the increased mobilization of proangiogenic cells. Also, the magnitude of attenuation on the Angpt/Tie-2 signaling axis after Arg administration in septic condition was comparable to the physiologic level of the normal control, which is suggestive that this signaling axis may play a crucial role in effectively resolving the inflamed vascular endothelium and attenuating remote lung injury. Whether the observed effect of Arg on enhanced proangiogenic cell mobilization and attenuated Angpt-Tie 2 signaling is beneficial in the amelioration of remote lung injury under condition of gut-derived polymicrobial sepsis may need to be clarified. 

## Figures and Tables

**Table 1:** The expression of endothelial progenitor cell (EPC) mobilizing factors in plasma.

	CXCL-12	MMP-9	VEGF	TNF-α	NO
	ng/mL		pg/mL		μmol/L
NC	4.6 ± 0.3 *	22.0 ± 1.4 *	65.0 ± 6.8 *	1.8 ± 0.6 *	4.1 ± 0.5 *
SS24	18.0 ± 1.2	48.8 ± 4.7	92.1 ± 2.4	16.3 ± 1.9	11.3 ± 0.5
SA24	17.1 ± 1.1	33.4 ± 4.0 ^#^	108.8 ± 1.0 ^#^	10.5 ± 0.8 ^#^	38.8 ± 1.2 ^#^
SS48	21.2 ± 1.1	46.3 ± 2.0	112.6 ± 6.3	10.1 ± 1.9	8.9 ± 0.2
SA48	27.9 ± 1.6 ^#^	30.6 ± 1.4 ^#^	130.1 ± 3.4 ^#^	4.96 ± 0.6 ^#^	15.7 ± 0.7 ^#^

NC, normal control group; SS24, sepsis at 24 h with saline; SA24, sepsis at 24 h with arginine; SS48, sepsis at 48 h with saline; SA48 sepsis at 48 h with arginine. All data are representative of duplicate measurements (*n* = 8). Data are presented as the mean ± SD. * Significantly differs from the sepsis group. ^#^ Significantly differs from the SS group at the same time point (*p* < 0.05).

**Table 2:** Quantitative level of cytokines protein expression in lung homogenates.

	IL-1	IL-6	TNF-α	IL-10	TGF-β1
	pg/mg				
NC	10.3 ± 1.2 *	12.5 ± 2.1 *	20.7 ± 2.3 *	265.8 ± 17.9	16.4 ± 1.8
SS24	92.6 ± 4.5	1275.6 ± 18.3	80.4 ± 3.4	235.8 ± 4.1	12.8 ± 1.6
SA24	44.3 ± 2.6 ^#^	662.3 ± 13.5 ^#^	42.9 ± 1.9 ^#^	367.8 ± 9.2 ^#^	55.3 ± 2.6 ^#^
SS48	83.2 ± 5.4	834.3 ± 17.3	67.8 ± 3.6	214.2 ± 5.6	16.6 ± 5.6
SA48	32.3 ± 2.3 ^#^	385.4 ± 12.8 ^#^	38.8 ± 3.2 ^#^	229.4 ± 7.7	22.4 ± 3.4

NC, normal control group; SS24, sepsis at 24 h with saline; SA24, sepsis at 24 h with arginine; SS48, sepsis at 48 h with saline; SA48, sepsis at 48 h with arginine. All data are representative of duplicate measurements (*n* = 8). Data are presented as the mean ± SD. * Significantly differs from the sepsis group. ^#^ Significantly differs from the SS group at the same time point (*p* < 0.05).
